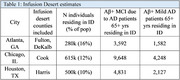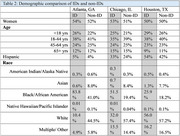# Geographic disparities in infusion center access for early Alzheimer's disease patients in the US

**DOI:** 10.1002/alz70858_101136

**Published:** 2025-12-25

**Authors:** Shardae Showell, Cai Gillis, Lauren Powell

**Affiliations:** ^1^ Biogen, Cambridge, MA, USA

## Abstract

**Background:**

Recent advances in Alzheimer's disease (AD) treatment include approval of two amyloid directed immunotherapies administered via intravenous infusion. These therapies require access to infusion centers, making availability of such facilities critical for effective treatment delivery. We examined accessibility of infusion centers across three cities in the United States. Our analyses aimed to identify patients with limited access to treatment and assess whether these gaps align with known disparities in Alzheimer's disease.

**Method:**

Three large metropolitan areas (Atlanta, GA; Chicago, IL; Houston, TX) with ethnic (>20% Hispanic/Latino) and/or racial diversity (>45% non‐white) were selected to examine characteristics of residents living in infusion deserts (IDs). The National Infusion Center Association Locator Data was used to examine gaps in availability at the neighborhood/census tract level with an ID defined as an area where the closest infusion center >5 miles radius. Demographic characteristics were compared between IDs and non‐IDs using CDC PLACES data.

To estimate the number of 65+ years amyloid‐positive (Aβ+) MCI due to AD and mild AD individuals in an infusion desert, we applied estimates of MCI/AD prevalence, AD severity, and amyloid abnormality to the estimates of those 65+ living in an ID.

**Result:**

Across three US metropolitan areas, approximately 12% of the population live in an ID. Among people 65+ years, approximately 18k living with Aβ+ MCI due to AD and 8k living with Aβ+ mild AD resides in an ID in one of these three cities (Table 1).

IDs showed a higher proportion of Hispanic/Latino residents compared to non‐IDs, ranging from 1% higher in Atlanta to 12% higher in Houston. A higher proportion of Black/African American individuals and a lower proportion of White and Asian individuals reside in an ID compared to non‐ID areas. Differences in the proportion of Black/African American race between ID and non‐IDs ranged from 7.7% higher in ID for Houston to 42.8% higher in Atlanta (Table 2).

**Conclusion:**

Approximately 26k individuals 65+ years with Aβ+ early AD are living in an ID across Atlanta, Chicago, and Houston. Locations of IDs are disproportionately Black/African American and Hispanic/Latino compared to non‐IDs, suggesting potential inequalities in access to new treatments for AD.